# Transcriptomic analysis of tigecycline-induced colistin collateral sensitivity in carbapenem-resistant *Enterobacter cloacae* complex

**DOI:** 10.1128/msphere.00903-25

**Published:** 2026-03-11

**Authors:** Youtao Liang, Jiming Wu, Jisheng Zhang, Chunli Wei, Jianmin Wang, Wenzhang Long, Xueke Jiang, Yang Yang, Mingjing Liao, Xuemei Gou, Xiaoli Zhang

**Affiliations:** 1 Department of Microbiology, the Affiliated Yongchuan Hospital of Chongqing Medical University 12550, Chongqing, China; University of Michigan, Ann Arbor, Michigan, USA

**Keywords:** *Enterobacter cloacae *complex, collateral sensitivity, colistin, tigecycline, transcriptomics

## Abstract

Utilizing treatment strategies based on collateral sensitivity (CS) represents a promising approach to suppressing antibiotic resistance. Although the mechanism of CS between numerous drugs has been researched, the mechanism of CS between tigecycline and colistin remains unclear. Therefore, the purpose of our research is to investigate the possible mechanism by which tigecycline affects colistin CS in the *Enterobacter cloacae* complex. Tigecycline induction significantly reduced the minimum inhibitory concentration of carbapenem-resistant *Enterobacter cloacae* complex (CRECC) to colistin, and sequencing revealed a single-base deletion at the RamR binding site. Complementation experiments demonstrated that deletion of the RamR binding site increased the resistance of CRECC417 to colistin and tigecycline by 2-fold and 4-fold, respectively. Transcriptomic comparison analysis of strains before and after CRECC417 induction revealed a total of 1,977 genes with significant differences in expression. Genes associated with carbohydrate, amino acid, and inorganic ion metabolism were the most highly enriched. Furthermore, the observed increase in colistin susceptibility in CRECC417R can be attributed to the inhibition of quorum sensing and biofilm formation pathways, as well as increased expression levels of genes associated with lipopolysaccharide biosynthesis and modification.

**IMPORTANCE:**

Due to the overuse of antibiotics, antimicrobial resistance (AMR) has become a serious threat to global public health. Dosage regimens based on bacterial CS can reduce antibiotic use without reducing efficacy, thereby reducing antibiotic-related toxicity risks, expanding the scope of antibiotic application, and limiting the development of antibiotic resistance. In this study, we analyzed the drug resistance mutations and global transcriptional changes in CRECC after tigecycline induction through genomics and transcriptomics. Our study showed that tigecycline exposure significantly inhibited quorum sensing pathways and biofilm formation. There were significant changes in the transcriptional levels of genes related to cell membrane lipopolysaccharide synthesis and modification, but no mutations were found in genes related to colistin resistance. These findings provide valuable insights for further investigation into the CS between tigecycline and colistin.

## INTRODUCTION

*Enterobacter cloacae* complex (ECC) is a group of common hospital pathogens belonging to the genus *Enterobacter* in the family Enterobacteriaceae, widely distributed in the natural environment (1). As a common opportunistic pathogen, ECC can cause a range of hospital-acquired infections, such as bacteremia, endocarditis, pneumonia, lower respiratory tract infections, and urinary tract infections (2). Clonal outbreaks of ECC members have been reported in various hospitals and regions, with ECC infections most commonly reported in clinical departments, particularly neonatal wards (3–5). Carbapenem antibiotics have long been considered the last resort for treating multidrug-resistant Gram-negative bacilli isolates. However, the widespread and inappropriate use of antibiotics has led to a steady increase in the resistance rate of Enterobacteriaceae to carbapenem antibiotics (6, 7). Due to the spread of carbapenemase-producing Enterobacteriaceae, carbapenem-resistant *Enterobacter cloacae* complex (CRECC) has become the third most common carbapenem-resistant Enterobacteriaceae (CRE) in China, following *Klebsiella pneumoniae* and *Escherichia coli* (8). Due to the ongoing development of antibiotic resistance and the insufficient research and development of new antibiotics, there is a growing need to use last-resort antibiotics, such as colistin and tigecycline (9–11).

Colistin and tigecycline are widely used in clinical practice as the last line of defense against CRE. Unfortunately, with the widespread use of colistin and tigecycline, resistance to these drugs has rapidly emerged worldwide. In recent years, resistance to colistin and tigecycline in Enterobacteriaceae has been frequently reported (12–14). Colistin belongs to the polymyxin family and is a polycationic antimicrobial peptide. Therefore, bacteria increase their resistance to colistin by modifying lipopolysaccharide (LPS) lipid A. Different strains of Enterobacteriaceae may have different mechanisms of resistance to colistin, but most of them usually modify lipid A with 4-amino-4-deoxy-L-arabinose (L-Ara4N) and/or phosphoethanolamine (PEtN) (15). Tigecycline, as a member of the tetracycline derivatives, reversibly binds to the 16S rRNA in the 30S subunit of the ribosome, blocking tRNA entry into the A site and inhibiting protein transcription and translation processes, thereby inhibiting bacterial growth (16). Reduced susceptibility to tigecycline is typically associated with the overexpression of multidrug resistance efflux pumps, such as AcrAB-TolC, OqxAB, and AdeABC (17, 18). The rise in antibiotic resistance has led to a dwindling number of antibiotics available for clinical use. Therefore, we urgently need new therapeutic strategies to address the antibiotic resistance crisis. A promising treatment strategy is to use collateral sensitivity (CS) to combine, sequence, or cycle (alternate) two antibiotics to curb the development of bacterial resistance (19).

CS refers to the phenomenon whereby increased resistance to one antibiotic increases the sensitivity of bacteria to another antibiotic. Lázár et al. first mapped the CS interaction networks of various antibiotics through large-scale laboratory evolution (20). Additionally, the mechanisms of CS have been extensively explored, such as the crucial role of reduced proton motive force (PMF) in the CS between aminoglycoside antibiotics and other antibiotics in *E. coli* and *K. pneumoniae* (20, 21). Plasmid-mediated expression of the β-lactamase gene *bla*_OXA-48_ induces CS to colistin and azithromycin, and this phenomenon is prevalent in Enterobacteriaceae (22). We have previously induced CS between tigecycline and colistin in CRECC and increased the bactericidal effect of colistin-resistant CRECC through combination therapy (23). However, the mechanism of CS between tigecycline and colistin has not been fully explored. Therefore, in this study, we induced CS to colistin with tigecycline *in vitro* and revealed the potential relationship between the CRECC resistance mechanism and CS through genomic and transcriptomic analyses. The aim was to provide a theoretical basis for designing treatment regimens based on the CS mechanism of colistin and tigecycline.

## MATERIALS AND METHODS

### Clinical strains and antimicrobial susceptibility testing

From December 2018 to April 2023, 212 clinical isolates of ECC were isolated from a teaching hospital. Based on the results of clinical susceptibility testing, a total of 38 CRECC strains were identified (24). Two colistin-resistant tigecycline-sensitive strains (CRECC401 and CRECC417) and 10 highly colistin-resistant CSECC strains were used in this study (Table S1). The broth microdilution method was used to determine the minimum inhibitory concentration (MIC) of antimicrobial drugs (tigecycline, colistin, meropenem, ertapenem, amikacin, ceftriaxone, ceftazidime/avibactam). *E. coli* ATCC 25922 and *Pseudomonas aeruginosa* ATCC 27853 were used as reference strains for quality control. The results were interpreted according to the breakpoints established by the European Committee on Antimicrobial Susceptibility Testing (http://www.eucast.org/clinical_breakpoints/). The antimicrobial susceptibility breakpoints for ECC were as follows: for colistin, susceptible ≤2 mg/L and resistant ≥4 mg/L; for tigecycline, susceptible ≤0.5 mg/L and resistant >0.5 mg/L.

### Antibiotic induction experiments and resistance stability experiments

Induce CRECC401 and CRECC417 in the presence or absence of tigecycline. Two monoclonal cultures of CRECC strains were incubated overnight in Luria-Bertani (LB) broth at 37°C with shaking at 180 rpm. Subsequently, 30 μL of the bacterial culture was added to 3 mL of fresh LB medium containing antibiotics at half the MIC concentration. The mixture was then incubated at 37°C with shaking at 180 rpm for 24 h. The antibiotic concentration was then doubled, and the cultures were continued under the same conditions for a total of 7 days. The strains were preserved throughout the induction process. The strains on the last day of induction were named CRECC401R and CRECC417R. CRECC401R and CRECC417R were passaged continuously for 3 days in antibiotic-free medium to test the stability of resistance. Ten CSECC strains that were highly resistant to colistin were induced with tigecycline for 7 days using the same method.

### Efflux pump inhibition assays

Efflux pump inhibitors (EPIs), such as carbonyl cyanide chlorophenol (CCCP; 16 mg/L) and phenylalanine-arginine-β-naphthylamine (PAβN, 20 mg/L), were used to assess efflux pump function. Determine the MIC value of tigecycline with or without EPI, using the standard broth microdilution method. Compared to the absence of EPIs, the MIC values of tigecycline were reduced by at least 4-fold with the addition of EPIs, which was considered to be a significant inhibition of efflux pumps (25).

### Detection of antibiotic resistance genes

Genomic DNA was extracted using the TIANamp Bacterial DNA Kit (TianGen Biotechnology, Beijing, China) according to the manufacturer’s recommendations. Polymerase chain reaction (PCR) was performed using the primers specified in Supplementary (Table S2). The amplified products were sent to Sangon Biotech (China) for Sanger sequencing, and the sequences were then sent to the Basic Local Alignment Search Tool (BLAST) for alignment analysis.

### RT-qPCR

Bacteria were inoculated onto blood agar plates and incubated at 37°C for 18 h. The bacterial suspension was adjusted to a McFarland turbidity of 2.3–2.5 using sterile saline. One milliliter of the bacterial culture was taken and centrifuged at 12,000 × *g* at 4°C to collect the bacterial pellet for RNA extraction. According to the manufacturer’s instructions, total RNA was extracted using the RNAprep Pure Bacterial Total RNA Extraction Kit (TianGen). The PrimerScript RT Kit (Japan Highland) was used for reverse transcription on each RNA sample. The relative expression levels of genes were quantified using SYBR Green Detection Reagent (MedChemExpress) on the CFX96 Fluorescent Quantitative Polymerase Chain Reaction System (Bio-Rad). Relative gene expression levels were calculated using the 2^-ΔΔCT^ method, and the results were normalized using *rpoB* as the reference gene for sample comparison. Each sample was subjected to three independent replicates to obtain the corresponding expression results. Table S2 lists the primer sequences used.

### Promoter knockout and complementation

As described earlier, the suicide vector homologous recombination method was used to construct strains with *ramA* promoter deletions and complementation mutants (26). Since CRECC417 is resistant to chloramphenicol, the chloramphenicol resistance gene on pRE112 was first replaced with the kanamycin resistance fragment from pCas9 via PCR. Next, 500 bp homologous arms upstream and downstream of the *ramA* promoter were PCR-amplified and fused into the suicide plasmid pRE112 via overlapping PCR. The recombinant plasmid carrying the fused DNA fragment was then thermally transformed into *E. coli* S17-1λpir, followed by transfer of the recombinant plasmid into *Enterobacter cloacae* via conjugation. Single recombinant colonies were selected on LB agar containing kanamycin (50 mg/L) and tetracycline (10 mg/L) for the first homologous recombination. Subsequently, the transformants were spread on LB agar containing 10% sucrose for the second homologous recombination. Finally, positive colonies were verified by PCR and sequencing. The primer sequences for constructing the mutant are shown in Table S2.

### RNA extraction, library construction, and sequencing

Total RNA was extracted from bacterial cells. The concentration and purity of the extracted RNA were detected using a Nanodrop 2000. RNA integrity was assessed by agarose gel electrophoresis, and the RIN value was determined using an Agilent 2100. The requirements for a single library construction were a total RNA amount of 2 μg, a concentration of ≥100 ng/µL, and an OD260/280 ratio between 1.8 and 2.2. Use the RiboCop rRNA Depletion Kit for Mixed Bacterial Samples (Lexogen, USA) to remove rRNA. Randomly fragment the mRNA into small fragments of approximately 200 bp. Using the mRNA as a template, perform reverse transcription with random primers to synthesize double-stranded cDNA. When synthesizing the second strand of cDNA, dUTP is used instead of dTTP for synthesis. The synthesized double-stranded cDNA is added to the End Repair Mix to complete the flat ends, with the 5′ end phosphorylated and an A base added to the 3′ end, followed by the attachment of a Y-shaped sequencing adapter. Then, the UNG enzyme is used to eliminate the second strand of cDNA containing dUTP, resulting in a library that only contains the first strand of cDNA. RNA library construction was performed using the Illumina Stranded mRNA Prep, Ligation kit (Illumina, San Diego, CA). RNA-seq paired-end sequencing is conducted using the Illumina NovaSeq Xplus.

### Bioinformatics processing and analysis

Bioinformatics analysis was performed using data generated by the Illumina platform. Gene expression levels were calculated using RSEM (https://deweylab.github.io/RSEM/) and measured using the TPM method. Differential expression analysis between samples was performed using the DESeq2 software (http://bioconductor.org/packages/release/bioc/html/DESeq2.html). Functional annotation and classification of differentially expressed genes (DEGs) were performed using the COG database (http://www.ncbi.nlm.nih.gov/COG/). Perform KEGG PATHWAY enrichment analysis using the Python scipy package (https://scipy.org/install/), with calculations conducted using Fisher’s exact test. To control the false positive rate, *P*-values were corrected using four multiple testing methods (Bonferroni, Holm, Sidak, and false discovery rate). Generally, when the corrected *P*-value (Padjust) ≤ 0.05, the data are considered statistically significant.

### Biofilm formation assay

Biofilm formation assays were performed in 96-well polystyrene microtiter plates, as previously described with some modifications (27). In brief, isolates were grown overnight in LB broth at 37°C. Subsequently, the culture was adjusted to 0.5 McFarland using sterile saline, then diluted 1:100 in LB medium. The control group was only added LB medium, and each sample was subjected to four biological replicates. The microtiter plates were incubated at 37°C for 24 h. The cell suspensions were then removed, the plates were washed once with 1× phosphate-buffered saline (PBS), and heat-fixed at 60°C for 15 min. Add 200 μL of 0.1% crystal violet (CV) solution to the wells and stain for 15 min. Remove excess CV and wash the wells three times with 1× PBS. Add 200 μL of 95% ethanol and dissolve for 15 min. Finally, read the absorbance of each well at 595 nm using a multifunctional microplate reader (Thermo Scientific).

## RESULTS

### Tigecycline induces CS to colistin

We screened two colistin-resistant strains, CRECC401 and CRECC417, from 38 CRECC strains. According to our previous study (24), both strains of bacteria belong to *Enterobacter kobei*. After 7 days of continuous induction with tigecycline *in vitro*, the MIC of colistin was significantly reduced. The MIC of CRECC401 decreased from 32 to 4, an 8-fold reduction, but colistin was still at a resistant level (Table 1). Compared to our previous studies (23), the MIC of the CRECC401 parental strain to colistin has decreased, which may be attributed to the attenuation of its resistance following multiple passages. The MIC of CRECC417 decreased from 16 to 1, a reduction of 16-fold (Table 1). After 7 days of culture without antibiotics in the control group, there was no significant change in the MIC of colistin for CRECC401 and CRECC417. After 3 days of continuous passage of the resistant strains in LB medium without antibiotics, CRECC417R remained sensitive to colistin, while CRECC401R regained resistance to colistin (Table 1). We further assessed the susceptibility of the relatively stable CRECC417R to other antibiotics. Interestingly, we found that the susceptibility of CRECC417R to ceftazidime/avibactam, imipenem, and ceftriaxone also significantly increased, with MIC values decreasing by 4-fold. The MIC values for ertapenem and amikacin decreased by 2-fold (Table 2).

**TABLE 1:** MIC values for colistin during tigecycline induction

Strain	COL MIC^*a*^ (mg/L)						
	Parental	2 mg/L TGC	4 mg/L TGC	8 mg/L TGC	16 mg/L TGC	Control	Passage 3 days
CRECC401	32	32	8	8	4	32	64
CRECC417	16	16	1	1	1	16	2

^*a*^ Minimum inhibitory concentration of colistin (COL).

**TABLE 2:** Changes in MIC of CRECC417 induced by TGC against different antibiotics^*a*^

Strain	MIC (mg/L)						
	CAZ-AVI	ETP	AMK	IPM	CRO	TGC	COL
CRECC417	0.5^S^	4^R^	2^S^	1^S^	32^R^	0.5^S^	16^R^
CRECC417R	0.125^S^	2^R^	1^S^	0.25^S^	8^R^	16^R^	1^S^

^*a*^ The superscript character “S” indicates that the MIC value is at a sensitive level, and the superscript character “R” indicates that the MIC value is at a resistant level. CAZ-AVI, ceftazidime/avibactam; ETP, ertapenem; AMK, amikacin; IPM, imipenem; CRO, ceftriaxone; TGC, tigecycline; COL, colistin.

### Deletion of RamR binding sites increases the MIC of CRECC for colistin and tigecycline

**Fig 1:**
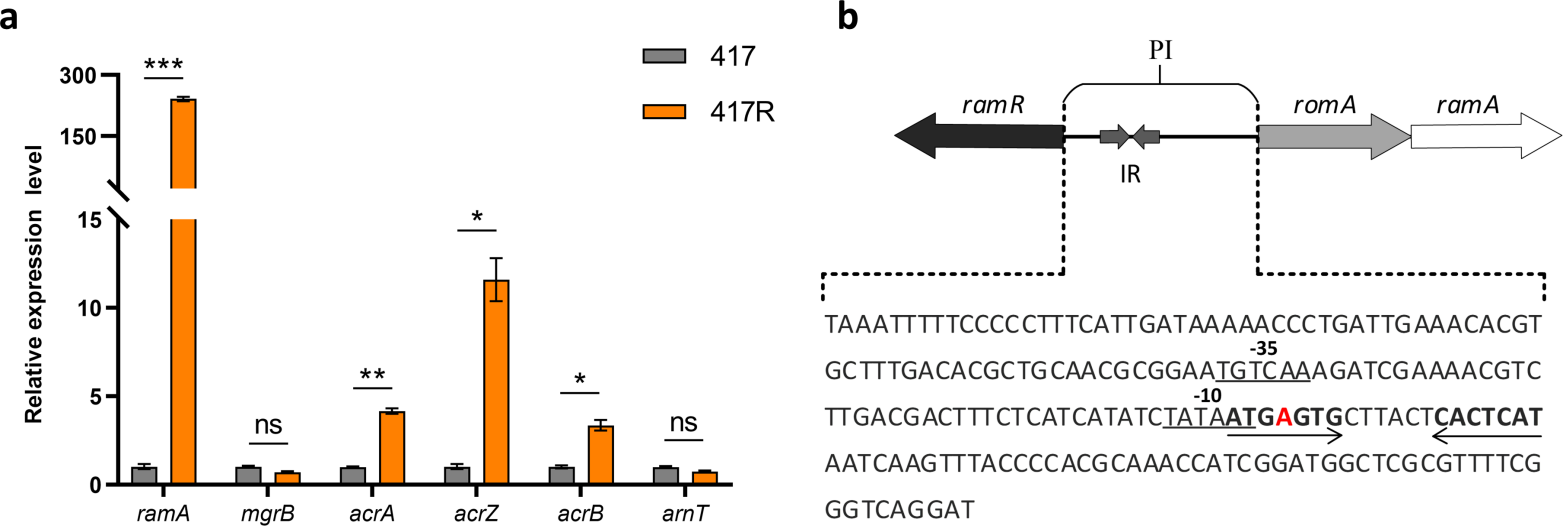
(**a**) Expression levels of genes associated with resistance to tigecycline and colistin. Data were normalized to the reference value of the *rpoB* gene. Statistical significance was assessed using unpaired *t*-tests and Welch’s correction, with *P* < 0.05 considered statistically significant; *P < 0.05, **P < 0.01, ***P < 0.001. ns, not significant. (**b**) Genetic structure of the *ramR-romAramA* locus. RamA is typically transcribed under the control of the PI promoter. RamR can bind to inverted palindromic sequences to inhibit RamA transcription. The inverted palindromic sequences are highlighted in bold and indicated by arrows. Base deletions are marked in red.

To investigate the molecular mechanism of tigecycline exposure leading to colistin sensitivity, we first evaluated the effect of efflux pumps through efflux pump inhibition experiments. The addition of PaβN reduced the MIC of CRECC417R to tigecycline by fourfold (Table 3). RT-qPCR also showed that the expression levels of genes associated with the efflux pump acrAB-tolC were significantly increased after induction, with the expression levels of *acrA*, *acrB*, and *acrZ* increasing by 4.1-fold, 3.3-fold, and 11.6-fold, respectively. The transcription regulator *ramA*, which regulates the expression of the efflux pump, also showed significant upregulation, increasing by 241-fold (Fig. 1a).

**TABLE 3:** MIC values for tigecycline in the presence or absence of EPIs

Strain	TGC MIC^*a*^ (mg/L)		
	NO	CCCP	PAβN
CRECC417	0.5	0.5	0.5
CRECC417R	16	16	4

^*a*^ Minimum inhibitory concentration of tigecycline (TGC).

Resistance gene mutation detection was performed for colistin resistance genes (*mgrB*, *phoP*, *phoQ*) and tigecycline resistance genes (*ramA*, *ramR*, *marA*, *marR*, *soxS*, *soxR*, *acrA*). Alignment revealed no mutations in the above genes, but we found a base A deletion in the PI promoter region of *ramA*, and this deletion was located in the reverse repeat sequence of the RamR binding site (Fig. 1b). To verify whether this deletion affects the MIC changes of tigecycline and colistin, we constructed a deletion mutant in 417. After knocking out the entire promoter, there was no change in the MIC of colistin, but the MIC of tigecycline was reduced by 2-fold. Complementation with the wild-type promoter did not change the MICs of colistin and tigecycline. However, complementation with the mutated promoter increased the MIC of colistin by 2-fold and the MIC of tigecycline by 4-fold (Table 4).

**TABLE 4:** CRECC417 promoter mutation on MIC values for colistin and tigecycline^*a*^

Antibiotic	MIC (mg/L)			
	CRECC417	CRECC417ΔPI	CRECC417ΔPI::PI	CRECC417ΔPI::PIR
COL	16	16	16	32
TGC	0.5	0.25	0.5	2

^*a*^ PI, *ramA* promoter; PIR,* ramA* promoter containing mutations.

### COG functional classification and KEGG enrichment analysis

**Fig 2:**
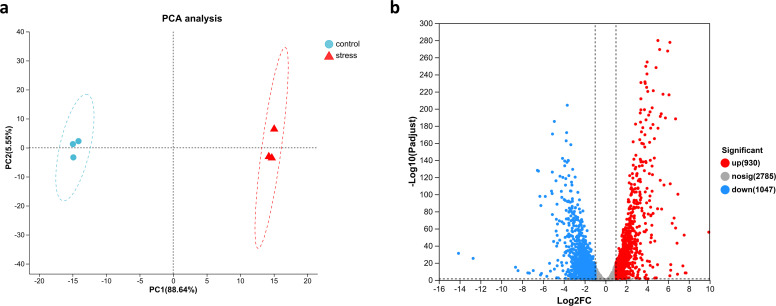
(**a**) Principal component analysis (PCA) of transcriptomic data shows distinct clustering among the two groups. The three points represent three replicates. Control refers to the group without tigecycline treatment, and stress refers to the tigecycline-induced group. (**b**) The volcano plot shows the fold change and significance of genes significantly affected by tigecycline relative to the control group (fold change ≥ 2, Padjust < 0.05).

**Fig 3:**
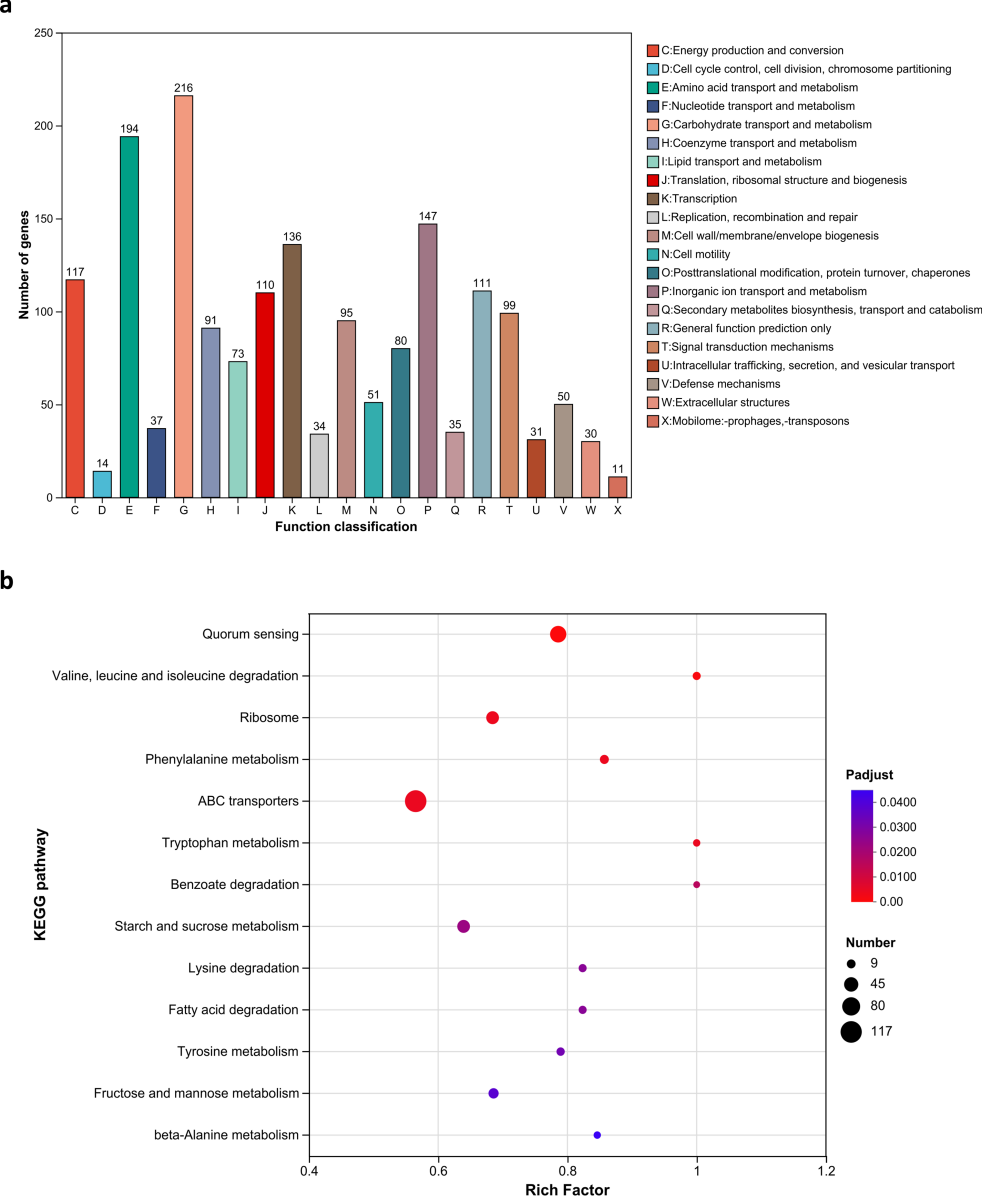
COG functional classification (**a**) and KEGG enrichment (**b**) of DEGs induced by tigecycline (fold change ≥ 2, Padjust < 0.05).

To further investigate the mechanism of CS to colistin after tigecycline induction of 417, we analyzed the global transcriptomic changes after induction using RNA-seq. Principal component analysis (PCA) showed significant differences between treated and control groups after tigecycline induction (Fig. 2a). A total of 1,977 genes were differentially expressed compared with the control group, of which 930 genes were upregulated and 1,047 genes were downregulated (Fig. 2b). COG functional classification revealed that carbohydrate transport and metabolism contained the highest number of DEGs, followed by amino acid transport and metabolism and inorganic ion transport and metabolism (Fig. 3a). The KEGG functional enrichment analysis showed that a total of 13 pathways were significantly enriched (Padjust < 0.05), of which the five most significantly enriched pathways were quorum sensing (QS), valine, leucine, and isoleucine degradation, ribosomes, phenylalanine metabolism, and ABC transporters (Fig. 3b).

### Tigecycline induction significantly inhibits QS and biofilm formation

Analysis of gene expression levels revealed that 55 out of 66 differential genes involved in the QS pathway were significantly downregulated (Table S3). SdiA (suppressor of cell division inhibitor) is involved in QS mediated by AHL signaling molecules (28, 29). Tigecycline exposure increased the expression of the transcriptional regulator *sdiA* by 2.2-fold. The AI-2/LuxS QS system is also widely present in Gram-negative bacteria and regulates a variety of bacterial physiological processes (30). The LuxS protein catalyzes the synthesis of the autoinducer-2 (AI-2) signaling molecule by 4,5-dihydroxy-2,3-pentanedione, and the expression of its encoding genes increased 3.8-fold. However, the expression of the *lsrACDBFG* operon, which is responsible for AI-2 uptake and activation, was reduced by 30.1-, 18.9-, 13.6-, 6.3-, 2.9-, and 6.4-fold, respectively (Table S3). The expression of proteins encoded by the *lsrRK* operon, which are involved in the regulation of the transcription of *lsrACDBFG*, was also reduced by 15.1-fold and 11.4-fold, respectively.

**Fig 4:**
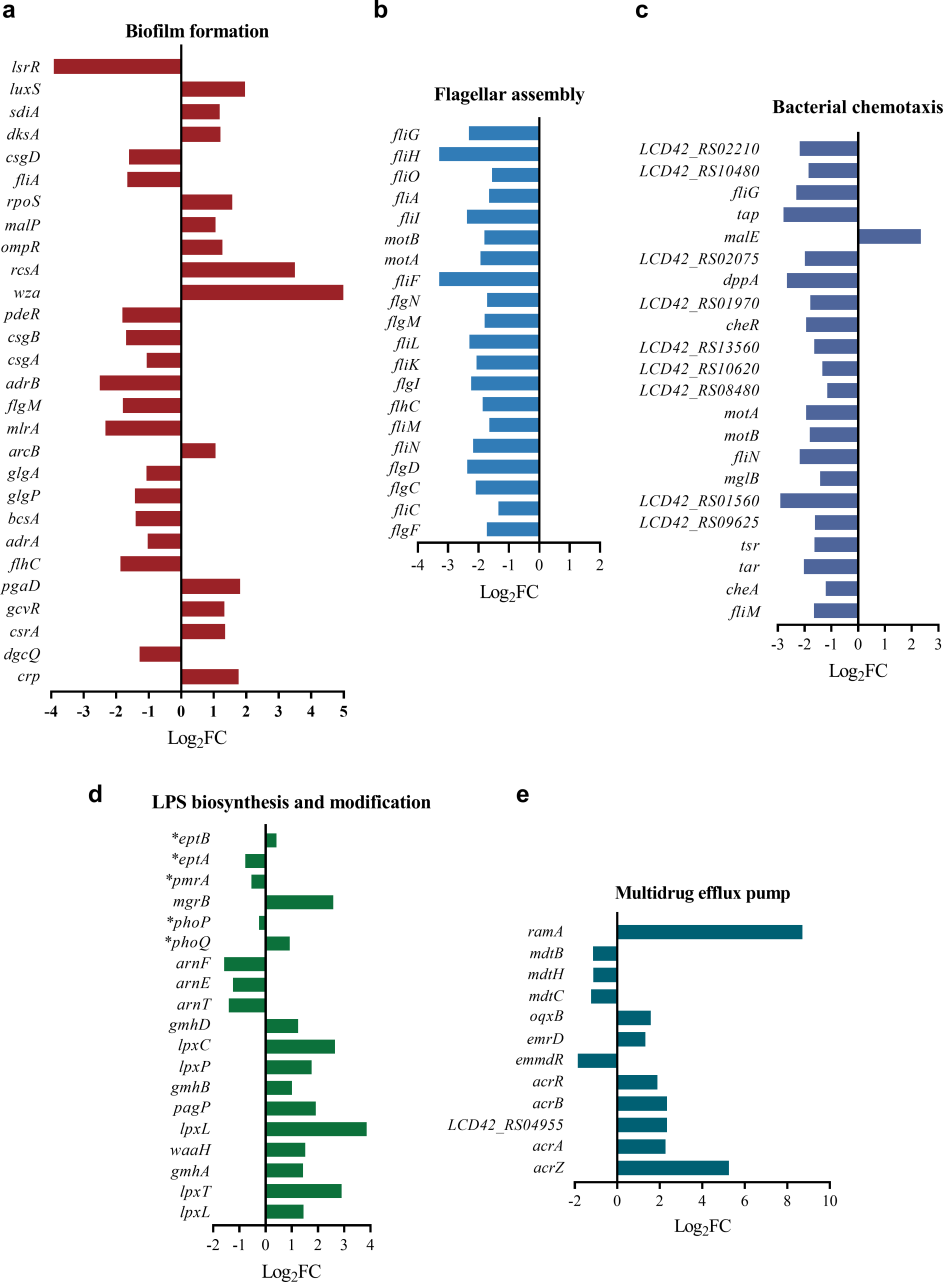
Expression patterns of DEGs associated with biofilm formation (**a**), flagellar assembly (**b**), bacterial chemotaxis (**c**), LPS biosynthesis (**d**), and multidrug resistance efflux pump (**e**). LPS, lipopolysaccharide. *, genes without significant differences.

In addition, 28 genes encoding biofilm formation pathways were significantly disordered (Fig. 4a). Flagella, cellulose, curli fimbriae, colanic acid, and EPS are essential for bacterial biofilm formation (31, 32). The expression levels of curli fimbriae synthesis genes *csgA*, *csgB*, and *csgD* were reduced by 2.1-fold, 3.2-fold, and 3.0-fold, respectively. The expression level of the cellulose biosynthesis gene *bcsA* was also reduced by 2.6-fold (Fig. 4a). However, the expression of *wza* and *pgaD*, which are associated with colanic acid biosynthesis and EPS formation, increased by 31.8-fold and 3.5-fold, respectively (Fig. 4a). The expression levels of all 20 DEGs associated with flagellar assembly were reduced (Fig. 4b), and of the 22 genes associated with bacterial chemotaxis, 21 genes were downregulated (Fig. 4c). Biofilm formation assays demonstrated that the biofilm-forming capacity of CRECC417R was significantly reduced (*P* < 0.01)(Fig. S1).

### Transcriptomic changes associated with LPS biosynthesis and modification

Transcriptome results showed that 11 genes involved in LPS biosynthesis and modification were upregulated (Fig. 4d). Among these, *lpxT* encodes LpxT kinase, which participates in the phosphorylation of lipid A (33), and its expression increased by 7.4-fold. Furthermore, the expression of *arnT*, *arnE*, and *arnF* was significantly reduced by 2.6-fold, 2.3-fold, and 2.9-fold, respectively (Fig. 4d). The L-Ara4N protein encoded by these genes can modify lipid A and reduce the negative charge of the cell membrane, thereby mediating resistance to colistin (15).

### Tigecycline reduces the MIC of colistin in high-level colistin-resistant ECC

To determine whether tigecycline induction can increase the sensitivity of high-level colistin-resistant *Enterobacter cloacae* to colistin, we induced 10 carbapenem-sensitive *Enterobacter cloacae* complex (CSECC) strains with tigecycline. Ten CSECC strains were highly resistant to colistin (MIC ≥ 128), and two of them contained *mcr-9* (24). After 7 days of continuous tigecycline induction, the MIC of eight strains decreased by ≥4-fold, the MIC of four strains decreased to a sensitive level, the MIC of three strains decreased from ≥128 to 8, and the MIC of one strain decreased to 32. Only two strains exhibited a change in MIC for colistin that was less than or equal to 2-fold. All 10 strains were resistant to tigecycline after induction, with MIC values ranging from 8 to 32 (Table S1). Biofilm formation assays revealed that among the eight strains exhibiting significantly reduced MICs for colistin, seven strains demonstrated significantly diminished biofilm formation capacity following induction (*P* < 0.05) (Fig. S1). Interestingly, although the MIC of CSECC4018 showed no significant change, its biofilm formation capacity was also significantly reduced (*P* < 0.0001).

## DISCUSSION

CS is a trade-off in the evolution of bacterial resistance. Trade-offs occur when adaptations enhance one trait at the expense of another (34). Bacteria typically increase their resistance levels through resistance gene mutations, and different resistance mutations can have opposite side effects. Some may increase the bacteria’s sensitivity to a second drug (CS), while others may increase resistance to other antibiotics (collateral resistance; CR) (35). Therefore, mutation analysis is commonly used in studies of the molecular mechanisms of CS. Several studies have also reported the role of drug resistance mutations in CS (36, 37). However, in our study, we did not detect mutations in the common resistance genes for colistin and tigecycline in the resistant mutant strain. Instead, we identified a base deletion in the promoter region of *ramA* (Fig. 1b). Alignment revealed that CRECC417 shares the same RamR protein-binding inverted repeat sequence as *K. pneumoniae* in the *ramA* promoter region (38), and that the deletion of this base is located within the inverted repeat sequence. Baucheron S et al. reported that a 2 bp deletion at the RamR binding site in *Salmonella enterica* serovar Typhimurium reduced RamR’s inhibitory effect on the *ramA* promoter, thereby activating the expression of the AcrAB-TolC efflux system, which plays a key role in the MDR phenotype (39). Consistent with previous studies, RT-qPCR revealed that the acrAB efflux pump in the drug-resistant mutant strain was activated (Fig. 1a). Additionally, the complementation mutants of the parental strain also exhibited increased resistance to colistin and tigecycline (Table 4). To our knowledge, this is the first time that the effect of RamR binding site mutations on resistance has been described in ECC.

**Fig 5:**
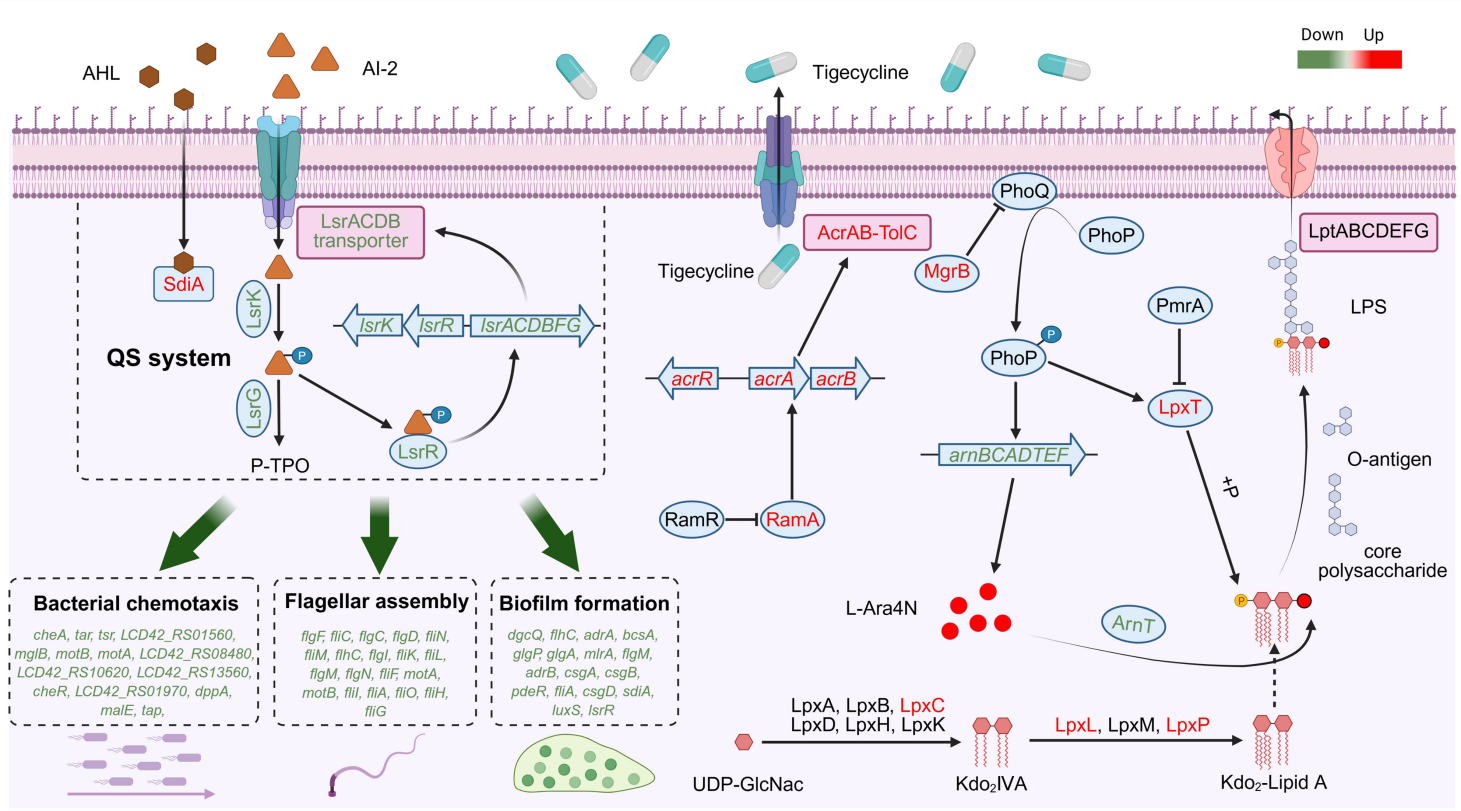
Transcriptional response diagram of carbapenem-resistant *Enterobacter cloacae* complex 417 (CRECC417) induced by tigecycline. (Created with BioRender.com.)

However, mutations in the RamR binding site did not cause the CS phenotype. This prompted us to perform transcriptomic sequencing to analyze the transcriptomic changes induced by tigecycline in CRECC417. Transcriptome results showed that tigecycline exposure significantly inhibited QS pathways and biofilm formation (Fig. 4b ; Table S3). This may lead to increased sensitivity to colistin, as biofilm formation has been shown to increase bacterial resistance to antibiotics. SdiA is an important LuxR-type signal molecule receptor in *Enterobacter cloacae* that binds to AHL signal molecules to regulate QS pathways (Fig. 5), and its expression increased 2.2-fold. Previous studies have shown that overexpression of *sdiA* inhibits bacterial biofilm formation, cell adhesion, and motility (28, 29). Similarly, our results also found that the expression levels of 15 genes involved in biofilm formation were significantly reduced. Genes related to cell adhesion (curli fimbriae) and motility (flagellar assembly and bacterial chemotaxis) were downregulated, and these phenotypes were all associated with biofilm formation (31, 32). In addition, biofilm formation experiments confirmed that the biofilm formation ability of CRECC417R was reduced after tigecycline exposure (Fig. S1). LuxS synthesizes the AI-2 signaling molecule involved in regulating the QS pathway, and its expression increased by 3.8-fold. However, the ABC transporters (LsrACDB) responsible for transporting AI-2 are encoded by the lsr operon, and their expression was significantly reduced. This may lead to a decrease in AI-2 uptake and utilization (Fig. 5). The *lsrRK* operon is located upstream of lsr, and its expression was also reduced. Within cells, AI-2 is phosphorylated by the cytoplasmic kinase LsrK to form an activated molecule (Fig. 5), which binds to LsrR and inactivates it, leading to increased transcription of the lsr operon (40). Phosphorylated AI-2 is further degraded by LsrF to 3,4,4-trihydroxy-2-pentanone5-phosphate (P-TPO) (41, 42). Studies have shown that the deletion of several genes in the lsr operon leads to defects in biofilm formation (43). In addition, the deletion of *lsrB* also leads to a decrease in *E. coli* chemotaxis (43, 44). Therefore, we speculate that tigecycline exposure causes biofilm formation defects by inhibiting the QS pathway, thereby increasing antibiotic sensitivity. Biofilm formation assays further corroborated that a majority of strains with reduced colistin MICs exhibited significantly diminished biofilm-forming capacity (seven of eight strains) (Fig. S1). Notably, strain CSECC4018 showed markedly reduced biofilm formation despite displaying no significant change in colistin MIC, suggesting that impairment of biofilm formation may not be a prerequisite for tigecycline-induced CS to colistin.

Transcriptome results showed that 11 genes involved in LPS biosynthesis were upregulated, among which *lpxC/L/P* and *lpxA/B/D/H/K* jointly participated in the synthesis of the LPS precursor Kdo_2_-lipid A (Fig. 5). Kdo_2_-lipid A then binds to core oligosaccharides on the inner membrane surface to form core-lipid A. Core-lipid A subsequently binds to O-antigen on the periplasmic surface of the inner membrane to form mature LPS. Finally, mature LPS is transported from the periplasmic side of the inner membrane to the outer membrane surface by ABC transporters (LptA-G) (45, 46). It has been reported that LPS loss increases resistance to colistin (47). Additionally, stimulating the activity of LptB (an LPS transport protein) can increase the susceptibility of Gram-negative bacteria to polymyxin (48). Therefore, we hypothesize that upregulation of LPS biosynthesis may lead to susceptibility to colistin; however, this requires further confirmation through lipidomics. Furthermore, consistent with our previous studies, disrupted expression levels of lipid synthesis genes were observed in strains exhibiting CS to colistin. However, these alterations lacked uniformity. Such variations may be associated with the distinct resistance mechanisms selected by bacteria during adaptation to antibiotic stress.

In Gram-negative bacteria, bacteria usually reduce the negative charge on the cell membrane surface through L-Ara4N and PEtN modification of lipid A (Fig. 5), resulting in colistin resistance. The *arnBCADTEF* operon is involved in the synthesis and transport of L-Ara4N, and the expression levels of *arnT*, *arnE*, and *arnF*, which are related to L-Ara4N transport, were significantly reduced by 2.6, 2.3, and 2.9 times, respectively (Fig. 4d). However, there was no significant change in the mRNA level of the PEtN transferase gene (*eptA*). Our previous studies have shown that, in *Enterobacter cloacae*, L-Ra4N modification mediated by the Arn/PhoPQ system leads to higher levels of colistin resistance than PEtN modification (49). Therefore, we speculate that the reduction in the transcriptional level of the arn operon after induction may increase the sensitivity of CRECC417 to colistin by reducing the positive charge modification of LPS. On the other hand, LpxT catalyzes the phosphorylation of lipid A at position 1, forming 1-diphospho lipid A, which increases the negative charge on the bacterial surface (50, 51). Sequencing results showed that the expression level of *lpxT* increased by 7.5-fold (Fig. 4d). This may further increase the negative charge on the bacterial surface after induction. Interestingly, *phoP* and *pmrA* are involved in the activation and inhibition of *lpxT*, respectively. Transcriptome results showed that the expression level of *phoP* increased by 1.9-fold, while that of *pmrA* decreased by 1.4-fold (Fig. 4d), which may explain the overexpression of *lpxT*, although this difference was not significant.

We used tigecycline to induce 10 CSECC clinical isolates that were highly resistant to colistin (MIC ≥ 128). The results showed that eight of the 10 CSECC strains had a ≥4-fold reduction in MIC for colistin, including two strains carrying *mcr-9* (4036 and 4043), which showed 16-fold and 64-fold reductions, respectively (Table S1). This means that even ECC with different resistance backgrounds can produce CS between tigecycline and colistin. However, two strains (4018 and 4034) showed no significant change in MIC, which requires further study to confirm the stable CS mechanism in different resistance backgrounds. CS-based treatment strategies provide new ideas for responding to the antibiotic resistance crisis and improving drug efficacy. However, the uncertainty of bacterial resistance evolution, different genetic backgrounds, and different environmental conditions or host environments may lead to CS instability and pose challenges to clinical application (52). Therefore, elucidating the mechanism of CS of tigecycline to colistin in different mutation contexts is of great significance for drug guidance and clinical practice.

### Conclusion

In conclusion, our study shows that the deletion of RamR binding sites increases the resistance of ECC to antibiotics. Tigecycline induces CS to colistin in CRECC, accompanied by significant transcriptional changes. Inhibition of QS pathways and biofilm formation, along with changes in the expression levels of LPS synthesis and modification genes, may explain the reduction in colistin MIC after tigecycline exposure. Future studies should further investigate the mechanisms by which tigecycline induces CS to colistin through molecular biology and lipidomics approaches. Additionally, expanding sample sizes is necessary to explore factors influencing the CS between tigecycline and colistin in different antimicrobial resistance contexts.

## Data Availability

The raw sequence reads (accession: PRJNA1305368) have been uploaded to the National Center for Biotechnology Information.

## SUPPLEMENTAL MATERIAL

Supplemental material msphere.00903-25-s0001.docx Tables S1 and S2 and Fig. S1.

Table S3 msphere.00903-25-s0002.xlsx Differential genes in quorum sensing systems.

ASM does not own the copyrights to Supplemental Material that may be linked to, or accessed through, an article. The authors have granted ASM a non-exclusive, world-wide license to publish the Supplemental Material files. Please contact the corresponding author directly for reuse.
