# Supplementary material for: Transcriptomic analysis of tigecycline-induced colistin collateral sensitivity in carbapenem-resistant Enterobacter cloacae complex
Source: mSphere. 2026 Mar 11;11(3):e00903-25. doi: 10.1128/msphere.00903-25 (PMC13037404; doi:10.1128/msphere.00903-25)
Supplement: Supplemental material — Tables S1 and S2 and Fig. S1. [file msphere.00903-25-s0001.docx]

**Table S1** Clinical isolates of ECC and changes in MIC values for colistin and tigecycline before and after tigecycline induction.

| Isolates | Species | COL MIC^a^(mg/L) | | TGC MIC^b^(mg/L) | | mcr9^c^ | NCBI accession numbers |
| --- | --- | --- | --- | --- | --- | --- | --- |
|  |  | Before induction | After induction | Before induction | After induction |  |  |
| 401 | *Enterobacter kobei* | 32 | 4 | 0.5 | 16 | - | JAMYDI000000000 |
| 417 | *Enterobacter kobei* | 16 | 1 | 0.5 | 16 | - | JBDYDE000000000 |
| 4001 | *Enterobacter asburiae* | ≥128 | 32 | 0.5 | 16 | - | JBDYDP000000000 |
| 4003 | *Enterobacter asburiae* | ≥128 | 2 | 0.5 | 8 | - | JBDYDQ000000000 |
| 4018 | *Enterobacter roggenkampii* | ≥128 | ≥128 | 0.5 | 16 | - | JBDYEF000000000 |
| 4024 | *Enterobacter kobei* | ≥128 | 0.25 | 0.5 | 32 | - | JBDYEK000000000 |
| 4034 | *Enterobacter kobei* | ≥128 | 64 | 0.5 | 16 | - | JBDYET000000000 |
| 4036 | *Enterobacter kobei* | ≥128 | 8 | 0.5 | 32 | + | JBDYEV000000000 |
| 4043 | *Enterobacter asburiae* | ≥128 | 2 | 0.5 | 16 | + | JBDYEZ000000000 |
| 4050 | *Enterobacter kobei* | ≥128 | 1 | 0.5 | 32 | - | JBDYFE000000000 |
| 4070 | *Enterobacter kobei* | ≥128 | 8 | 0.5 | 32 | - | JBDYFU000000000 |
| 4072 | *Enterobacter cloacae* | ≥128 | 8 | 0.5 | 32 | - | JBDYFW000000000 |

**^a^**Minimum inhibitory concentration of colistin (COL).

**^b^**Minimum inhibitory concentration of tigecycline (TGC).

**^c^** *mcr-9* carriage status. -, does not carry *mcr-9*; +, carries *mcr-9*.

**Table S2** Primers used in this study.

| Primers | Sequence (5' → 3') |
| --- | --- |
| Primers for PCR | |
| *mgrB*-F | GTCCAATCCAGAGTATCGCC |
| *mgrB*-R | ACTCACCCATTTCACCACCTC |
| *phoP*-F | GGTCGACGAGCTGAAGTAACAC |
| *phoP*-R | GAAGCTGACGCTGTAACCCAC |
| *phoQ*-F | GGGCGTTTGCGTAAGAAAATTCAG |
| *phoQ*-R | TATTCCGCAGGTTCTTACTGACAC |
| *ramA*-F | TCGGGTGAGTGATACCACAC |
| *ramA*-R | TGGTTTTCACTGGCGTCCTG |
| *ramR*-F | TTTGACATTCCGCGTTGCAG |
| *ramR*-R | CCGACCTTGAAGACGTCGTAC |
| *marR*-F | CGGCTGACTCATTCAGTTGACT |
| *marR*-R | CGATCCAGTCCAAAATGCTATG |
| *marA*-F | AGATGAAGTGGCAACCCTTGAG |
| *marA*-R | GCTGACGGCAAAATCATCGT |
| *soxS*-F | GGAATTATACTCGCCCGCAG |
| *soxS*-R | TGGCACTTCGCGAAAGCGAT |
| *soxR*-F | TGCTGATGCGACATAAGGTT |
| *soxR*-R | CAGCGGGATAGAGTGAAAGAC |
| Primers for RT-qPCR | |
| *acrZ*-F | CATCATCCTCGGTGCCATCT |
| *acrZ*-R | TGCTGCTTTTTAGCCTGGTC |
| *mgrB*-F | ACATCCTGATCGCACATCAC |
| *mgrB*-R | ACGCTGGGTGATTCTGATTGT |
| *ramA*-F | GAATATAGCGCCCCAGGCTT |
| *ramA*-R | CATTGAAGACATTGCCCGCC |
| *acrA*-F | TCACCAGCTCCACTTTGGCT |
| *acrA*-R | TTGACGTAACCCAGTCCAGC |
| *acrB*-F | TCCGTCACTTCATCCAGCAC |
| *acrB*-R | CAAGCTCATTCCTGCCTGAT |
| *arnT*-F | CAAAGGTTTTCTGGCGCTGG |
| *arnT*-R | AGCCAGCCAAAAATCAGCAC |
| *rpoB*-F | AAGGCGAATCCAGCTTGTTCAGC |
| *rpoB*-R | TGACGTTGCATGTTCGCACCCATCA |
| Primers for gene editing | |
| Chl-F | TTTAGCTTCCTTAGCTCCTGAAAATCTCG |
| Chl-R | GGCGTTGGCCTCAACACGATTTTACGTC |
| Kan-F | TCAGGAGCTAAGGAAGCTAAAATGAGCCATATTCAACGGG |
| Kan-R | CGTGTTGAGGCCAACGCCTTAGAAAAACTCATCGAGCATC |
| H1-F | CTTCCCCGTTTCCCGCATGTGGTGGCAAACTCCATCGTGC |
| H1-R | GATGAAGAGAGTTTTTTTCATGTGGCACGTCCGAAGAGTG |
| H2-F | CACTCTTCGGACGTGCCACATGAAAAAAACTCTCTTCATC |
| H2-R | GCGCTCGTCACCATTTGGGAGTCGTAGTGATCGTGAGAG |
| pRE112-F | CACATGCGGGAAACGGGGAAGGTCAAGTTC |
| pRE112-R | CTCCCAAATGGTGACGAGCGCATAGCCGG |
| single-exchange F | TGTCCATCGCTCTCCTCCTG |
| single-exchange R | ATGAGGCTGCGGTTGCGTTC |
| double-exchange F | GTTGAGGCGGCAATACCTG |
| double-exchange R | CCGGCATTCAGAACAAACGG |


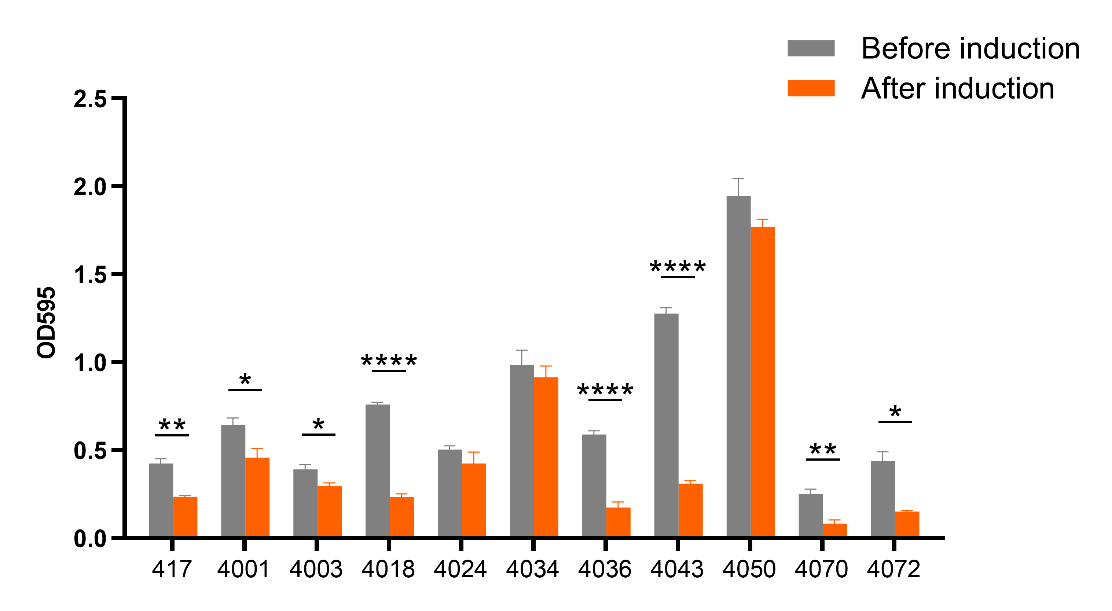


**Fig. S1** The ability of the induced strains to form biofilms was assessed after 24 hours of cultivation in 96-well microtiter plates containing LB broth. Differences between groups were analyzed using unpaired t-tests and Welch's correction. * p < 0.05, ** p < 0.01, *** p < 0.001, **** p＜0.0001.
